# Molecular clocks in ancient proteins: Do they reflect the age at death even after millennia?

**DOI:** 10.1007/s00414-021-02522-1

**Published:** 2021-02-17

**Authors:** Nina Sophia Mahlke, Silvia Renhart, Dorothea Talaa, Alexandra Reckert, Stefanie Ritz-Timme

**Affiliations:** 1 Institute of Legal Medicine, University Hospital Düsseldorf, Heinrich Heine University, Düsseldorf, Germany; 2 Department of Archaeology & Coin Cabinet, Joanneum Universal Museum, Graz, Austria; 3 Regional Archaeology, Direction of the Museum “Das Dorf des Welan”, Wöllersdorf-Steinabrückl, Austria

**Keywords:** Molecular clocks, Age estimation, Aspartic acid racemization, Pentosidine, Ancient proteins

## Abstract

Age at death estimation in cases of human skeletal finds is an important task in forensic medicine as well as in anthropology. In forensic medicine, methods based on “molecular clocks” in dental tissues and bone play an increasing role. The question, whether these methods are applicable also in cases with post-depositional intervals far beyond the forensically relevant period, was investigated for two “protein clocks”, the accumulation of D-aspartic acid (D-Asp) and the accumulation of pentosidine (Pen) in dentine. Eight teeth of skeletons from different burial sites in Austria and with post-depositional intervals between c. 1216 and c. 8775 years were analysed. The results of age at death estimation based on D-Asp and Pen in dentine were compared to that derived from a classical morphological examination. Age at death estimation based on D-Asp resulted consistently in false high values. This finding can be explained by a post-mortem accumulation of D-Asp that may be enhanced by protein degradation. In contrast, the Pen-based age estimates fitted well with the morphological age diagnoses. The described effect of post-mortem protein degradation is negligible in forensically relevant time horizons, but not for post-depositional intervals of thousands of years. That means that the “D-Asp clock” loses its functionality with increasing post-depositional intervals, whereas Pen seems to be very stable. The “Pen-clock” may have the potential to become an interesting supplement to the existing repertoire of methods even in cases with extremely long post-depositional intervals. Further investigations have to test this hypothesis.

## Introduction

In forensic medicine, the identification of an unknown deceased is an important task and may be the key to solving a homicide. One of the main prerequisites for successful identification is the estimation of age at death. This is especially true for the investigation of skeletal finds, which is a field of intersection between forensic medicine and anthropology.

Also in anthropological casework with very long post-depositional intervals of up to many thousands of years, knowledge of the ages at death in a past population may be important—for example, if insights into demography, health and social conditions are of interest.

Classical methods for age at death estimation in human skeletons are based on morphological changes. Whereas morphological methods reveal mostly satisfactory results in the period of growth and development, they may be associated with much higher errors in adulthood [1–3]. The histomorphological approach based on tooth cementum annulation provides precise age estimates in some working groups [4–7]. Morphological age at death estimation may be difficult in incomplete, badly preserved skeletons.

In the last decade, molecular methods of age at death estimation have attracted much attention in forensic sciences. The usability of so-called molecular clocks like the “epigenetic clock” (based on DNA methylation) and “protein clocks” (accumulation of D-aspartic acid and pentosidine in permanent proteins) for age at death estimation has been explored by analysing samples from individuals with known ages, and their high potential in the forensic context (with short post-depositional intervals of up to c. 50 years) was confirmed [8–18]. A combination of different molecular clocks or a combination of morphological and molecular findings promise even better results and have been proposed as basis for the development of new, multivariate methods [8, 13, 19]. Molecular methods are also applicable to skeletons, since teeth and bone samples are suitable for these analyses [13, 20].

The question arises if such “molecular clocks” reflect the age at death even after millennia. This is only possible if molecules that relate to the relevant molecular clocks are present and intact in very old tissues. While the probability to extract enough intact DNA suitable for the analysis of DNA-methylation is very low in archaeological samples, proteins may be quite well preserved for a long time [21–23]. Dobberstein et al. (2008) reported a remarkable stability of the protein matrix of dentine; amino acid composition as well as the electrophoretic pattern after cyanogen bromide (CNBr) cleavage of collagen appeared unchanged in naturally aged human teeth with post-depositional intervals of up to 1700 years. CNBr patterns were remarkably consistent even in much older human bone samples up to c. 6000 years [22]. Therefore, the accumulation of D-aspartic acid (D-Asp) and the accumulation of pentosidine (Pen) as “protein clocks” might be an interesting tool for anthropological casework, too.

**Fig. 1:**
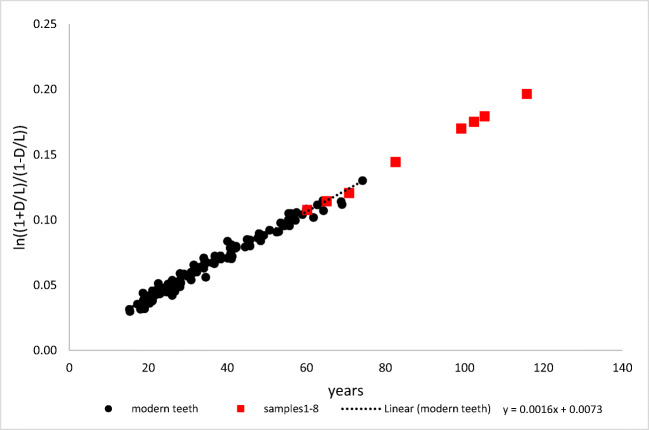
Content of D-aspartic acid (D-Asp, as ln((1+D/L)/(1-D/L))) in dentine of modern teeth of individuals with known ages (black dots; reference data, extended sample of third molars from Ritz-Timme (1999); *n*=118, *r*=0.99) and in the eight archaeological dentine samples (red squares). Estimation of the ages at death for the archaeological samples was based on the depicted reference data under consideration of the different times of root development (+ 9 years for first molars, + 4 years for second molars)

The accumulation of D-Asp in permanent proteins during lifetime is the result of a non-enzymatic conversion of L-asparagine residues and L-aspartic acid residues into their D-forms [24, 25, 18]. The data depicted in Fig. 1 illustrate the accumulation of D-Asp in dentine during lifetime. The relationship between D-Asp and age is very close and can be used as basis for age at death estimation. In forensic casework, age estimation based on the D-Asp content in dentinal protein is one of the most accurate methods for age at death estimation in adults [26, 27, 14, 16, 17]. The application of this method to archaeological samples has been less successful so far; this has been attributed to diagenetic changes [28–30].

**Fig. 2:**
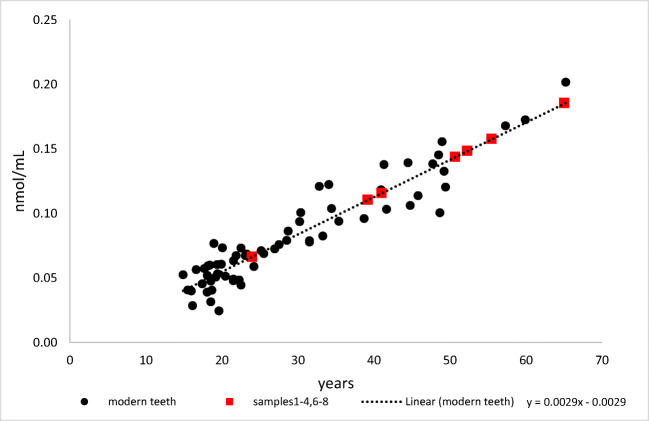
Content of pentosidine (Pen, as nmol/mL) in dentine of modern teeth of individuals with known ages (blue dots; reference data, sample set of third molars from Greis et al. (2018); *n*=63, *r*=0.94) and in seven archaeological dentine samples (red squares; sample 5 exhibited unquantifiable pentosidine concentrations). Estimation of the ages at death for the archaeological samples was based on the depicted reference data under consideration of the different times of root development (+ 9 years for first molars, + 4 years for second molars)

The “Pen-Clock” is based on the accumulation of pentosidine during lifetime. Pentosidine is an advanced glycation end product. Such protein modifications are the result of glycation, which is a non-enzymatic reaction of free amino groups (mainly of arginine and lysine) with glucose or with other reducing carbohydrates [31–33]. Pen accumulates in permanent proteins during lifetime in several tissues [34–36], among others also in dentine [10, 35]. The data presented in Fig. 2 illustrate the intravital accumulation of Pen in dentine. The analysis of Pen has been suggested for age at death estimation in a forensic context, especially in combination with other parameters and as part of multivariate models [8, 10]. So far, this molecular approach has not been tested in cases with long post-depositional intervals. If the “Pen-clock” is conserved in very old tissue, it may reflect age at death over millennia and could be an interesting supplement to the existing repertoire of methods for age estimation in anthropological casework.

The access to eight teeth from skeletons with post-depositional intervals up to c. 8775 years gave us the opportunity to explore the potential of the “Pen-clock” for the investigation of archaeological samples for the first time. Since there are only few data regarding the “D-Asp-clock” in very old tissues, we also analysed this parameter.

## Material and methods

Age at death estimation based on the D-Asp and Pen contents in dentine was performed by analysis of eight archaeological teeth with post-depositional intervals between c. 1216 and c. 8775 years. The resulting age estimates were compared to the results of age estimation based on morphological methods. Morphological and molecular age estimations were performed independently; the results were only disclosed after completion of all analyses.

### Archaeological teeth

Table 1 gives an overview of relevant information on the archaeological teeth.

**Table 1:** Information about the analysed eight archaeological teeth: tooth types, archaeological ages, burial sites, and taphonomically relevant information (*f* female, *m* male, ? most likely result of sex determination)

- No. - Sex - Tooth type	Archaeological age (years); Archaeological period	- Burial site - Taphonomically relevant information
Sample 1 F? First molar	1417–1517 (dating via archaeological finds) Late antiquity	- Mattersburg, tomb 334, Austria - Shaft grave partly with limestone quarry - Individual burial - Almost complete skeleton, teeth in situ
Sample 2 M Second molar	5110–5370 (radiocarbon dating) Neolithic	- Wöllersdorf, tomb 4744, skeleton 3/I, Austria - 60-cm deep grave pit, covered with rubble and large limestone blocks - Individual burial - Almost complete skeleton, teeth in situ
Sample 3 M First molar	1217–1317 (dating via archaeological finds) Early middle ages	- Mattersburg, tomb 339, Austria - Shaft grave partly with limestone quarry - Individual burial - Almost complete skeleton, teeth in situ
Sample 4 F Second molar	1216–1316 (dating via archaeological finds) Early middle ages	- Mattersburg, tomb 285/I, Austria - Shaft grave partly with limestone quarry - Individual burial - Almost complete skeleton, teeth in situ
Sample 5 M? Second molar	7005–7225 (radiocarbon dating) Mesolithic	- Pöttsching, object 1, skeleton 13, Austria - Partial pit, in sandy loam - Individual burial - Almost complete skeleton, teeth in situ
Sample 6 M First molar	1216–1316 (dating via archaeological finds) Early middle ages	- Mattersburg, tomb 261, Austria - Shaft grave partly with limestone quarry - Individual burial - Almost complete skeleton, teeth in situ
Sample 7 F Second molar	5207–5507 (radiocarbon dating) Neolithic/Chalcolithic	- Josephinengrotte/Peggau, Steiermark, Austria - Individual burial - Partly preserved skeleton, teeth in situ
Sample 8 M First molar	8835–8775 (radiocarbon dating) Mesolithic	- Wöllersdorf, Austria - 30 cm deep pit, “Quarzitgerät,” coarse-grained limestone gravel soil - Secondary burial - Skull (without mandible) and left femur diaphysis, bone surface weathered, teeth in situ

All skeletons were excavated in Austria. Their archaeological ages were between c. 1216 and 8775 years (in the four oldest samples according to radiocarbon dating, in the four samples from the early middle ages according to archaeological finds, as indicated in Table 1). They were mostly well preserved but partly fragmented and—with the exception of sample 8—(almost) complete; sample 8 comprised a skull and a left femur diaphysis. All teeth (first and second molars) were found in situ; they were healthy and complete.

Samples 1, 3, 4, and 6 (archaeological ages between 1216 and 1517 years, as indicated in Table 1) were excavated in Mattersburg, Ried Stückl, Austria, in an early medieval cemetery.

Sample 2 (archaeological age c. 5370–5110 years) comes from an excavation site in Wöllersdorf Satzäcker, Austria, from a multiple burial of 13 individuals from the late Neolithic era.

Sample 5 (archaeological age c. 7005–7225 years) was found at a rescue excavation in Pöttsching, Mattersburg, Austria, together with material from a chalcolithic Epi-Lengyel culture [37].

Sample 7 (archaeological age c. 5207–5507 years) was recovered from a cave (“Josefinengrotte”) in Peggau, Steiermark, Austria [38, 39].

The skull and the left femur diaphysis of sample 8 (archaeological age c. 8835–8775) were excavated during a research excavation in Wöllersdorf-Steinabrückl, Austria. This area has been continuously inhabited since the early Neolithic era. The skull was assigned to the Mesolithic era; it seems to be a secondary burial.

### Modern teeth

Modern teeth were donated with informed consent by living persons after tooth extraction as a result of medical procedures.

### Preparation of teeth

Root dentine was prepared according to Ritz-Timme (1999). The roots were cut from the crown, and the distal third of the roots, the cementum, and dental pulp were removed. The quality of preparation was checked using ultraviolet light at a wavelength of 366 nm (at this wavelength, only dentine appears bright bluish). After some washing steps (double distilled water, 15% sodium chloride, ethanol/ether (3:1; v/v), and 2% sodium dodecyl sulphate), the samples were lyophilised, pulverised, and stored at −20°C until further analysis.

### Analysis of the D-Asp content in dentine

D-Asp was analysed by gas chromatography (GC) as described by Ritz-Timme (1999). Ten milligram of each sample was hydrolysed in 1 mL 6 N hydrochloric acid (HCl) for 6 h at 100°C. For derivatisation, 1 mL isopropanol and 1 μL sulphuric acid were added to each sample, and the samples were heated at 110 °C for 1 h. Isopropanol was removed by drying using a nitrogen stream. After adding 1 mL 4 N ammonia solution and 1 mL dichloromethane, the samples were centrifuged, and the resulting two phases were separated and dried again. One milligram dichloromethane and 50 μL trifluoroacetic acid (TFAA) were added; the samples were then heated for 15 min at 60 °C and dried using a nitrogen stream. Separation and quantification of D- and L-aspartic acid were performed via GC on a chiral capillary column (Chirasil-L-Val, Varian). Each sample was analysed at least twice. D/L ratio was calculated by using the arithmetic mean. The D-Asp content was calculated as ln((1 + D/L)/(1 − D/L)).

### Analysis of the Pen content in dentine

The concentrations of Pen were determined by high-pressure liquid chromatography (HPLC), as described by Greis et al. (2018). Fifty milligram of each sample was hydrolysed in 1 mL 6 N HCl for 18 h at 110°C. The dried residues were dissolved in 1 mL 0.01 M heptafluorobutyric acid (HFBA), filtered through a syringe filter (0.45-μm pore diameter, 25-mm diameter, VW International) and dried. The samples were dissolved in 250 μL pyridoxine-HFBA (pyridoxine 2.068815 μmol/mL in 0.01 M HFBA) and then injected into the HPLC system (HPLC 1100 Series, Agilent, CA). The stationary phase was a semi-preparative column (Onyx™ Monolithic Semi-PREP C18, LC Column 100 × 10 mm, Phenomenex, CA). A linear gradient of 10–85 % acetonitrile (eluent B) and 0.1 % HFBA (eluent A) from 0 to 32 min was used as mobile phase; the flow rate was set to 1 mL/min. A wavelength of 335 nm was used for excitation and a wavelength of 385 nm for detection; Pen was identified by its retention time. The concentration of Pen was determined using a calibration curve. The lower limit of quantification was 0.06 nmol/L.

### Age at death estimation based on D-Asp and Pen

Age at death estimation based on D-Asp was performed according to the method of Ritz-Timme (1999). The data set of Ritz-Timme (1999) was extended by additional 18 third molar samples and served as reference data set (*n*=118, Fig. 1).

Age at death estimation based on Pen was carried out by the method of Greis et al. (2018); the data of Greis et al. (2018) served as reference data (Fig. 2).

Based on the reference data sets (modern teeth), the relationship between age and Pen or D-Asp (as ln(1+D/L(/(1-D/L) (Ritz-Timme 1999)) was described by linear regression analysis, and correlation coefficients were determined. Mean absolute errors (MAEs) for age estimates based on the D-Asp and Pen reference data (modern teeth) were calculated.

Dental molecular clocks do not reflect the age of an individual directly but the age of the analysed tooth. Therefore, age at death estimation based on D-Asp or Pen has to consider tooth type specific times of root development. Whereas the analysed archaeological teeth were first and second molars, the reference data (Figs. 1 and 2) were derived from third molars. The roots of first molars develop c. 9 years and the roots of second molars c. 4 years earlier than the roots of third molars [40, 41]. Therefore, the age estimates derived from the used reference data had to be corrected by +9 years for first molars and of +4 years for the second molars.

### Amino acid analysis

Amino acid analysis was performed by HPLC in all eight ancient dentine samples as well as in two modern dentine samples according to the method described by Dobberstein et al. (2008). In short, 10 mg of each sample was hydrolysed in 1 mL 6 N HCl for 24 h at 110 °C. The dried residues were dissolved in 400 μL 0.01 N HCl. Human collagen type I (Sigma-Aldrich/Merck KGaA, Darmstadt, Germany) served as external standard. OPA (o-phthaldialdehyde) reagent and FMOC (9-flourenylmethylchloroformate) were used for derivatisation of the primary, and secondary, respectively, amino acids. The stationary phase was a C18 column (Hypersil BDS, C18 250 × 3 mm, particle size 5 μm; Thermo Electron GmbH, Dreieich, Germany). The mobile phase consisted of eluents A (40 mM NaH2PO4, 1.5 mM sodium azide) and B (45% methanol, 45% acetonitrile, 10%H2O) according to Heems et al. [42]. The amino acid derivatives were detected over a period of 50 min using a binary gradient. The flow rate was set at 1.2 mL/min and the column temperature at 40 °C. A wavelength of 335 or 260 nm was used for excitation and a wavelength of 440 or 305 nm for detection of the OPA derivatives or the FMOC derivatives, respectively. The amino acids were identified by their retention times.

### Morphological age at death estimation

Morphological age at death estimation was performed by an experienced anthropologist (by S. Renhart) using anthropological standard methods [43, 44].

Morphological age estimation could be based on several parameters in all cases, but in sample 8, only the skull and the left femur diaphysis were available.

The following morphological approaches were applied (according to [45]), unless otherwise stated:

- Samples 1 and 5 (subadult):
  Dental development [46], epiphyseal ossification [43], and length of the long bones [47]
- Samples 2–4 and 6–7 (adult):
  Dental findings (degree of wear, dental attrition) [48], closure of cranial sutures [44, 49], facies symphysialis ossis pubis [49, 50], facies articularis sternalis clavicularis [51], and proximal ends of femur and humerus [52]
- Sample 8 (adult; isolated skull and a left femoral shaft):
  Dental findings (degree of wear, dental attrition) [48] and closure of cranial sutures [44, 49]

The age at death estimates presented in Table 2 were based on the synopsis of all parameters taking into account their variability [45].

**Table 2:** Age at death estimates based on D-aspartic acid (D-Asp) and pentosidine (Pen) analyses, as compared to the results of morphological age at death estimation in eight archaeological samples (PDI, post-depositional interval; n.d. too low Pen concentration, not detectable)

No. PDI (years)	D-Asp (years)	Pen (years)	Morphological methods (years)
Sample 1 1417–1517	74.26	13.97	15–18
Sample 2 5110–5370	99.76	47.65	51–70
Sample 3 1217–1317	97.47	58.08	51–70
Sample 4 1216–1316	56.27	35.31	25–35
Sample 5 7005–7225	67.20	n.d.	14–16
Sample 6 1216–1316	56.28	44.35	35–45
Sample 7 5207–5507	96.41	52.80	45–55
Sample 8 8835–8775	108.50	32.27	31–40

## Results

### Amino acid analysis

**Fig. 3:**
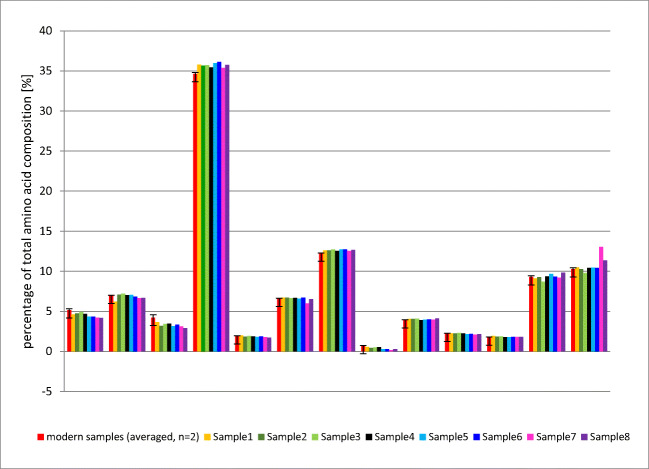
Amino acid composition of the eight archaeological dentine samples and two modern samples (ASX, asparagine and aspartic acid; THR, threonine; SER, serine; GLX, glutamine and glutamic acid; PRO, proline; HYP, hydroxyproline; GLY, glycine; ALA, alanine; VAL, valine; MET, methionine; ILE, isoleucine; LEU, leucine; TYR, tyrosine; PHE, phenylalanine; TRP, tryptophan; ARG, arginine)

Figure 3 depicts the results of the amino acid analysis of the eight archaeological dentine samples, as compared to the amino acid composition of two control samples from modern teeth. The analyses did not reveal relevant differences in the amino acid composition, neither between the archaeological and modern samples nor within the archaeological samples.

### Results of age at death estimation based on dentinal D-Asp and Pen contents, as compared to the morphological age diagnoses

Figures 1 and 2 depict D-Asp and Pen contents in dentine of modern teeth from individuals with known ages (reference data) and in the archaeological teeth.

The relationship between Pen or D-Asp (as ln(1+D/L(/(1-D/L) [20]) and age at death in the reference data sets can be described by the regression equations given in Figs. 1 and 2; the correlation coefficients were *r*=0.99 for D-Asp and *r*=0.94 for Pen. Based on the reference data (modern teeth), the MAEs of age at death estimation were calculated to be 2.23 years for D-Asp and 3.92 years for Pen.

Table 2 presents the results of age at death estimation based on the two “protein clocks” in the samples of the eight archaeological teeth, as compared to the results of morphological age at death estimation.

In seven samples, the age estimates based on the Pen content were highly compatible with the results of age estimation based on morphological methods. Sample 5 exhibited very low, unquantifiable pentosidine concentrations, fitting with the assumption of very young dentine (morphologically estimated age of death 14–16 years, see Table 2).

In contrast, age estimates based on the D-Asp content were in all cases—as compared to the morphological age diagnoses—far too high. There was no relationship between the deviation between the morphological or the molecular age estimates and the length of the post-depositional intervals.

## Discussion

The analysis of two molecular clocks revealed that only one clock (accumulation of pentosidine) may reflect the right age at death even after millennia, whereas the other clock (accumulation of D-aspartic acid) is massively disturbed by post-depositional processes over such a long time.

### Accumulation of D-aspartic acid

The age estimates based on the D-Asp content of the analysed archaeological teeth were implausibly high (Table 2, Fig. 1), obviously due to a further accumulation of D-Asp residues after death.

After death, the organism cools down to the surrounding temperature, and the conversion of L-amino acids into their D-form is slowed down extensively. An increase of the D-Asp content after death should be negligible within short post-depositional intervals of up to decades [53]; in forensically relevant cases, a relevant post-mortem increase of D-Asp is not to be expected under normal environmental conditions. However, the accumulation of D-Asp progresses slowly and plays a role in the analysis of archaeological samples [28]. The increase of D-Asp during the post-depositional interval may be enhanced by protein degradation. Whereas significant protein degradation is not to be expected within the first decades after death [23], very long post-depositional intervals may result in increasing protein degradation [54, 23]. Degradation of collagen and non-collagenous proteins of dentine may lead to small peptide fragments with low steric hindrances, resulting in a fast conversion of L-amino acid into their D-form [21–23].

The summary of amino acid composition of the analysed ancient total dentine samples did not show relevant differences, as compared to that of modern dentine samples (Fig. 3). Although this is the result of a remarkable stability of dentinal collagen [22, 23], it does not exclude a gradual degradation process with release of small peptide fragments from otherwise intact polypeptide chains [55, 23]. Griffin et al. (2009) assumed that released small peptides and free amino acids diffuse from dental hard tissue into the burial environment. However, the kinetics of such a leaching process is not clear. The dentinal matrix may retain released peptides for a certain time before they are leached into the environment. We analysed total tissue (and not purified collagen); that means that our samples included even all peptides entrapped in the dentinal matrix. The presence of small peptides and amino acids released from degraded dentinal protein and entrapped in the dentinal matrix may be a relevant factor for the accumulation of D-Asp in total dentine after death; this assumption is in line with the findings of [55, 22], who reported high D-Asp contents especially in the acid-extracted protein fraction of archaeological dentine samples.

The post-mortem accumulation of D-Asp further increases the D-Asp content that has already been developed during lifetime. The kinetics of this post-mortem accumulation is not predictable, since it depends on many factors such as temperature, pH, humidity, colonisation with microorganisms, or the degree of leaching. Even assuming that several individuals would have been buried under identical post-mortem conditions, only the differences between their ages at death could be further detectable (due to the preservation of intravital accumulated D-Asp), while the individual ages at death themselves could not be calculated since a reliable prediction of the post-mortem increase of D-Asp in a single case is not possible due to unpredictable differences in the microenvironment.

The D-Asp values in the eight samples were unrelated to the post-depositional intervals; this may be attributed to different kinetics of protein degradation as well as to different extents of retainment and leaching of released peptides and amino acids.

In conclusion, very long post-depositional intervals (high archaeological ages) may destroy the functionality of the “D-Asp clock” by protein degradation and leaching of released peptides and amino acids.

### Accumulation of pentosidine

Pen residues are located almost exclusively in the dentinal collagen, which remains remarkably stable over long post-depositional intervals [21–23]. Moreover, the formation of pentosidine results in complex protein structures due to cross-linking [56, 57]. It can be assumed that these glycation-induced changes stabilise the affected molecular region even more and protect it against degradation. If this is true, the “Pen-clock” may be preserved even over very long post-depositional intervals.

This assumption is supported by the fact that the results of the morphological age estimation by conventional anthropological methods agree well with the age estimates based on the “Pen-clock” in all cases (Table 2), with only one exception (sample 5).

## Conclusions

Whereas the “D-Asp-clock” may result in false high age at death estimates due to advanced protein degradation after long post-depositional intervals, the “Pen-clock” may obviously be conserved even over millennia. The good agreement between morphological age estimates and the age estimates derived from the dentinal Pen content in all samples (apart from one sample with very low, unquantifiable Pen content) is remarkable.

However, the significance of our results is limited by several facts. We analysed only eight well-preserved teeth from similar, relatively cool, and constant environments. The presented data do not allow conclusive statements regarding the potential of the “Pen clock” under various post-depositional conditions yet. Moreover, we have to be aware of the fact that we compared the molecular age estimates not with true ages but with morphological age estimates, which in turn may be associated with relevant errors.

Despite these limitations, the presented data indicate that the “Pen-clock” has the potential to become an interesting supplement to the existing repertoire of methods for age estimation in forensic and anthropological casework. It may be useful in the investigation of incomplete skeletons or isolated/fragmented teeth with limited morphological findings and/or a lack of applicability of morphological methods due to the bad condition of samples. In complete skeletons, a combination with morphological methods in multivariate models may enable more accurate age estimates by use of information from different biological levels [8].

In conclusion, the “Pen clock” needs and deserves further and systematic investigations, optimally using samples from archaeological contexts with known ages at death that were exposed to different environmental conditions. Since it is difficult to get access to such samples, we would be happy if this article leads to collaborations that enable further investigations.
